# Marine Health-Promoting Compounds: Recent Trends for Their Characterization and Human Applications

**DOI:** 10.3390/foods10123100

**Published:** 2021-12-14

**Authors:** Eva Quitério, Cristina Soares, Ricardo Ferraz, Cristina Delerue-Matos, Clara Grosso

**Affiliations:** 1 Ciências Químicas e das Biomoléculas/CISA, Escola Superior de Saúde—Instituto Politécnico do Porto, Rua Doutor António Bernardino de Almeida 400, 4200-072 Porto, Portugal; evaquiterio@hotmail.com (E.Q.); ricardoferraz@eu.ipp.pt (R.F.); 2 LAQV-REQUIMTE, Instituto Superior de Engenharia do Porto, Instituto Politécnico do Porto, Rua Doutor António Bernardino de Almeida 431, 4249-015 Porto, Portugal; cmm@isep.ipp.pt (C.D.-M.); claragrosso@graq.isep.ipp.pt (C.G.); 3 LAQV-REQUIMTE, Departamento de Química e Bioquímica Faculdade de Ciências, Universidade do Porto, R. do Campo Alegre, 4169-007 Porto, Portugal

**Keywords:** seaweeds, purification methods, biological activities, analytical methods

## Abstract

Seaweeds represent a rich source of biologically active compounds with several applications, especially in the food, cosmetics, and medical fields. The beneficial effects of marine compounds on health have been increasingly explored, making them an excellent choice for the design of functional foods. When studying marine compounds, several aspects must be considered: extraction, identification and quantification methods, purification steps, and processes to increase their stability. Advanced green techniques have been used to extract these valuable compounds, and chromatographic methods have been developed to identify and quantify them. However, apart from the beneficial effects of seaweeds for human health, these natural sources of bioactive compounds can also accumulate undesirable toxic elements with potential health risks. Applying purification techniques of extracts from seaweeds may mitigate the amount of excessive toxic components, ensuring healthy and safer products for commercialization. Furthermore, limitations such as stability and bioavailability problems, chemical degradation reactions during storage, and sensitivity to oxidation and photo-oxidation, need to be overcome using, for example, nanoencapsulation techniques. Here we summarize recent advances in all steps of marine products identification and purification and highlight selected human applications, including food and feed applications, cosmetic, human health, and fertilizers, among others.

## 1. Introduction

Macroalgae or seaweeds are a diverse group of multicellular photosynthetic organisms distributed worldwide in marine environments [1]. They are commonly classified into three taxonomic groups according to their chemical structure and pigmentation, namely brown algae (Ochrophyta), green algae (Chlorophyta) and red algae (Rhodophyta) [2].

Despite representing an abundant resource, the growing demand for these organisms and the concern regarding the impact of climate change on seaweed abundance, distribution, and quality, create the need to invest in algae cultivation and production strategies [3]. For these reasons, the farming of seaweeds has expanded rapidly, and in 2015, the global seaweed production totalled 30.4 million tons, with the naturally growing seaweed sector responsible for 1.1. million tons and the farmed sector for 29.4 million tons [4]. The three leading producers are China, Indonesia, and the Philippines, besides being the ones that cultivate the widest variety of seaweed species [3,5].

The most valuable cultivated seaweeds are *Saccharina japonica* (J.E. Areschoug) C.E. Lane, C. Mayes, Druehl & G.W. Saunders, *Undaria pinnatifida* (Harvey) Suringar, *Sargassum fusiforme* (Harvey) Setchell, *Porphyra* sp., *Euchema* sp., *Kappaphycus alvarezii* (Doty) L.M.Liao, *Gracilaria* sp., *Enteromorpha clathrata* (Roth) Greville, *Monostroma nitidum* Wittrock, and *Caulerpa* sp. [4], differing in the purpose of its production to the diverse intended applications.

Seaweed compounds have been used as gelling, thickening, and emulsifying agents in a wide variety of food products. Nowadays, marine algae are also identified as a source of biologically active compounds with beneficial effects on health, which amount is sensitive to changes in growing conditions such as water temperature, salt content, nutrients, and light [6].

Besides, seaweeds also contain a rich source of structurally diversified primary and secondary metabolites such as peptides, lectins, carotenoids, polysaccharides, fatty acids, flavonoids, and phytosterols, with great potential for application in both food, cosmetic and pharmacological industries, distinguishing themselves notably from terrestrial plants [7]. These compounds are responsible for many bioactivities, from antioxidant, antiviral, antifungal, antibacterial to antiproliferative, anti-inflammatory, adipogenesis, antidiabetic and neuroprotective [8].

**Figure 1:**
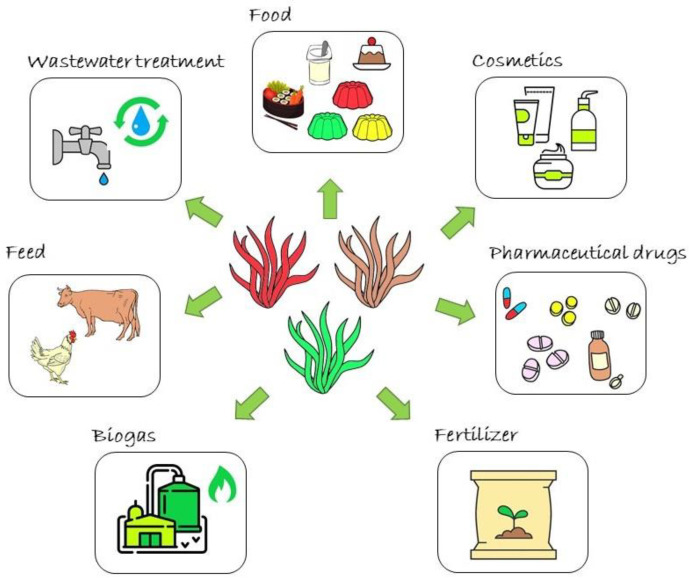
Human uses of seaweeds.

Therefore, this review aims to critically discuss the most applied purification processes and analytical methodologies available to characterize seaweed bioactive compounds as well as summarize the most promising human applications for seaweed raw materials and extracts (Figure 1).

## 2. Macroalgae as a Source of Biologically Active Compounds

Over the past decades, seaweeds’ nutritional value and health-promoting effects have been intensively studied due to the anti-inflammatory and antioxidant functions in diverse diseases, such as cancer, atherosclerosis, skin abnormalities, and neurodegeneration [7].

Besides their common use as gelling, thickening, emulsifying, and preserving ingredients in various food products [9]; nowadays, marine algae gained a tremendous interest as a source of bioactive compounds beyond all the food applications and the widely recognized good source of iodine [10].

It has been shown that seaweeds produce biologically active compounds against UV radiation, stress, and herbivores [6]. These compounds such as polysaccharides, phenols, carotenoids, phytosterols, proteins, bioactive peptides, omega-3 fatty acids, and tocopherols exhibit nutritional and health-promoting effects. Table 1 presents the proximate composition and the average content of total phenolic, carotenoid, tocopherol and phytosterol in green, red and brown seaweeds.

### 2.1. Polysaccharides

Polysaccharide properties from the marine environment, such as anticoagulant, antitumour, cancer preventive, antimicrobial, anti-inflammatory, and antioxidant, make them prospective healthy compounds with an extensive scope of applications [22]. Besides this, polysaccharides like agar, carrageenans and alginate produced by algae impart functional value to food. Furthermore, these polysaccharides can provide physical stability to emulsion systems due to their structure and physicochemical features dependent on the organism that produces them [6].

Agar is a dried amorphous gelatinous substance composed of a mixture of agarose and agaropectin [23], extracted distinctively from the genera *Gelidium*, *Gracilaria*, *Gelidiella*, *Pterocladia*, *Pterocladiella*, and *Ahnfeltiopsis* [24,25,26].

Likewise, carrageenans are water-soluble linear sulfated polysaccharides synthesized by species of red seaweeds [27]. It is a high-molecular-weight polysaccharide consisting of repeating galactose structures and 3,6 anhydrogalactose units, joined by alternating α-1–3 and β-1–4 glycosidic linkages [28]. The sulfate groups define the carrageenans by their number and position on repeating galactose units [26].

There are three major commercial classes of carrageenans, namely, iota, kappa, and lambda, that differ structurally and in their gelling properties, and thus in their uses [5,28]. Both kappa and iota carrageenan form gel with K and Ca salts [5]. Kappa forms a more robust, rigid, and elastic gel with K salts, while Ca produces a brittle and stiff gel form [28]. This type of carrageenan produces the strongest gels among all classes, although presenting the liability of being most susceptible to synaeresis (bleeding of liquid). However, bleeding can be reduced by blending iota and lambda carrageenans with the kappa type and adding locust bean gum [5]. Iota forms elastic gels with Ca salts more strongly than with K salts, being the first ones soft, resilient, virtually free of bleeding and with the peculiar characteristic of thixotropic flow, meaning that stirring the gel makes it flow like a thick liquid, but if left standing it will gradually return to its original state [5]. Lambda is non-gelling, creating high viscosity solutions [5].

Alginates are anionic linear polysaccharides extracted from cell walls of brown seaweed, comprised of mannuronic and glucuronic acid units, responsible for the flexibility of the seaweed [5,28]. Brown seaweeds genera primarily used to isolate alginates are *Ascophyllum*, *Ecklonia*, *Laminaria*, *Lessonia*, *Macrocystis*, and *Sargassum* [27].

Alginate composition varies from seaweed to seaweed [5]. For this reason, there are an extensive range of alginates with differing viscosities that can also be affected by extraction conditions, lowering it if conditions are too severe [5]. Generally, stronger gels are produced with glucuronic acid-rich alginates [5]. The most stable type is Na alginate, and the less stable is alginic acid [25].

**Figure 2:**
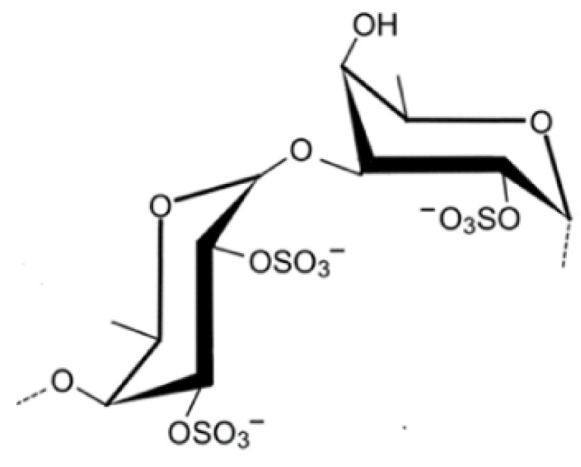
Example of a fucoidan structure.

Other major polysaccharides produced by seaweeds comprises fucoidan (sulfated polysaccharides) (Figure 2) and laminarans (non-sulfated polysaccharide) in brown algae, and ulvan in green algae [6,7,22]. Studies revealed that these compounds exhibit promising in vitro antioxidant activity, radical scavenging, and metal-chelating abilities [22]. However, the antioxidant activity of seaweeds polysaccharides is highly connected to their structural characteristics like the degree of sulfating, relative molecular mass, type of the dominant sugar, and glycosidic branching [9].

Fucoidan, from the homo- and heteropolysaccharides families, shows evidence of neuroprotective, antitumor, antiviral, and anticoagulant activities [7]. In this context, this bioactive compound has been shown to normalize the levels of some of the enzymes involved in Alzheimer’s disease [7], suggesting a therapeutic potential of fucoidans. Also, investigations have revealed that fucoidan use has reduced behavioural deficits, increased striatal dopamine and its metabolites levels, and reduced cell death, contributing to neuroprotection and enhancement of brain health, ameliorating neuron conditions often challenged by various toxic materials [29].

Generally, polysaccharides like fucoidan, phorphyran, furcellaran, and laminarin are extracted by applying acidic solvents or water and posteriorly precipitated to separate alginates using calcium chloride [6].

### 2.2. Polyphenols

Polyphenols are a class of natural organic compounds composed of multiples aromatic rings bonded directly to one or more hydroxyl groups. Polyphenols could be divided into several classes like flavonoids and tannic acid [30].

Polyphenols in algae are phlorotannins, such as phloroglucinol (1,3,5-trihydroxy benzene), bromophenols, phenolic acids, tannins, and flavonoids [6,7,29]. The high presence of phenolic compounds in algae is closely associated with antioxidants, reactive oxygen species (ROS) scavenging, and other biological activities [9]. These compounds play a critical function in cell defence against abiotic and biotic stress in algae, while in mammals, they act as free radical scavengers, reducing agents and metal chelators, and therefore successfully inhibiting lipid oxidation [9,31].

Phloroglucinol, abundant in the brown alga *Ecklonia cava* Kjellman was found to attenuate Aβ- induced ROS accumulation [7]. In addition, this natural compound revealed its potential action as an antioxidant by reducing Aβ- induced dendritic spine reduction and attenuating cognitive impairment [7].

**Figure 3:**
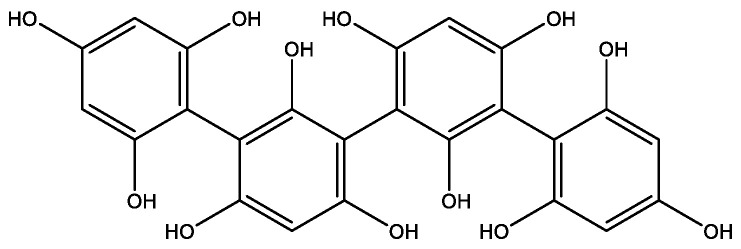
Tetrafucol A, a fucol-type phlorotannin found in the brown alga *Ascophyllum nodosum* (L.) Le Jolis.

There are phlorotannins associated with this compound, polymers of phloroglucinol units, classified according to the linkage nature between the monomers [6,22]. Characterized by diverse and abundant natural polyphenols, they are secondary metabolites exclusive to seaweeds [22]. Generally, localized in the physodes, they predominate, especially in brown algae (Figure 3) [6]. Molecular weights of these bioactive compounds vary from 0.126 to 650 kDa [22], and its content can go from 1 to 14% in different macroalgae [6]. Phlorotannins present several bioactivities such as antioxidant, antiproliferative, antibiotic, antidiabetic, anti-HIV, antiallergic, antineuroinflammatory and anti-inflammatory properties [6,29].

Another interesting class of bioactive phenols is bromophenols (BPs), commonly present in red algae [29]. Vidalols A and B are BPs that exhibit anti-inflammatory properties, shown by the potential ability to inhibit bee venom-derived phospholipase A2 (PLA2) activity [29]. PLA2 modulates the release of arachidonic acid and the generation of eicosanoids, potent inflammatory mediators [32]. Thus, strict regulation of PLA2 activity in the brain is vital since an instability of this well-balanced system induces oxidative stress and neuroinflammation, which may cause several neurological diseases [29]. Thus, these compounds reveal potential use as inhibitors of neuroinflammation.

In addition, dieckol, also a phlorotannin, scavenges ROS production in murine microglia (BV-2) cells, acting against neuroinflammation as well as other phlorotannins such as eckol, 7-phloroeckol, phlorofucofuroeckol A, and dioxinodehydroeckol [9,29].

As expected, the extraction methods profoundly affect seaweed extracts’ total phenolic content and antioxidant activity [6]. Although there is no exclusive and defined protocol for extracting phenolic compounds from algae, the most used method is solvent extraction, using both high polar and non-polar solvents [33]. For example, for phlorotannins, the traditional method uses ethanol or methanol as solvent, which requires further purification by high-pressure liquid chromatography or silica gel chromatography and characterization by nuclear magnetic resonance (NMR) techniques [6].

### 2.3. Carotenoids

Carotenoids are lipophilic pigments synthesized by photosynthetic bacteria, plants, fungi, and algae [34]. They consist of eight isoprenoid units joined to form a conjugated π system in the carotenoid skeleton, the conjugated polyene structure responsible for the typical colour of each carotenoid [35].

β-carotene, lutein, violaxanthin, neoxanthin, and zeaxanthin are found mainly in green algae, while red algae contain mainly α- and β-carotene, lutein, and zeaxanthin, and finally brown algae exhibit β-carotene, violaxanthin, and fucoxanthin [6].

These pigments have been of great interest due to their protective role against photooxidation in the abovementioned algae classes [22]. Furthermore, they reveal their importance in promoting human health and preventing chronic diseases due to their pro-vitamin A activity and antioxidant properties [35].

The human body cannot produce most of these substances independently, so they must be obtained through a diet rich in carotenoids, like fruit and vegetables [36]. In addition, they are an important antioxidant in seaweeds, demonstrating strong radical scavenging activity [9], which makes algae-derived carotenoids useful against oxidative stress-induced diseases [29]. However, studies indicated that carotenoids’ antioxidant activities depend on structure, location or site of action, and potential interaction with another antioxidant, concentration, and partial oxygen pressure [9].

**Figure 4:**
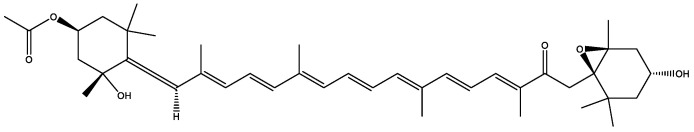
Chemical structure of fucoxanthin.

Among the carotenoids, β-carotene is considered one of the most important antioxidants. However, fucoxanthin, majorly present in brown seaweeds, is particularly interested due to its ability to modulate central nervous system-related processes [7]. Fucoxanthin, represented in Figure 4, is a xanthophyll with an unusual allenic bond and 5,6-monoepoxide in its molecule [22]. The ability to absorb light efficiently makes fucoxanthin an accessory pigment in the photosynthetic process [22]. This marine carotenoid shows antioxidant, anticancer, anti-obesity, antidiabetic, and antiphotoaging activity [6]. Besides the antioxidant activity, fucoxanthin reduces Aβ plaque formation and, together with β-carotene, prevents ROS formation indicating a potential role for fucoxanthin in treating Alzheimer’s disease [7]. Moreover, anti-inflammatory properties have been found in carotenoids, such as inhibiting the inflammatory response in macrophages [7].

Commonly, pigments from plant sources are extracted via solvent extraction using hexane as a non-polar solvent [6] and solid-phase extraction (SPE) using methanol [22]. Nevertheless, new and efficient technologies for extraction purposes have been applied, such as extraction using vegetable oils, supercritical fluid extraction, and pressurized liquid extraction [6].

### 2.4. Phytosterols

**Figure 5:**
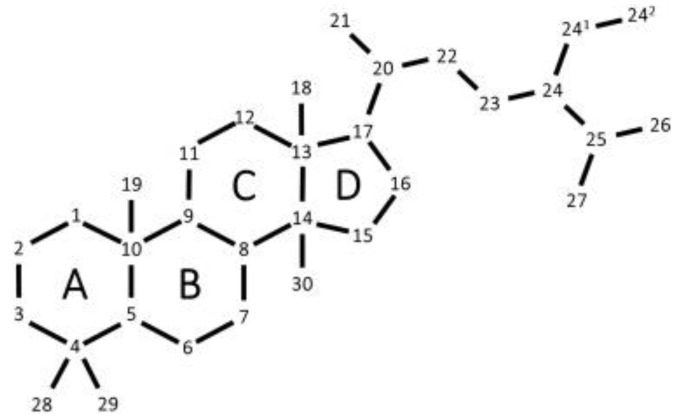
Schematic structure of the steroid skeleton.

Phytosterols are naturally occurring steroid alcohols found in plants and seaweeds, similar to cholesterol, but with extra ethyl or methyl group in the side chain [37]. Phytosterols comprise plant sterols and stanols, a saturated form of plant sterols (Figure 5) [38].

Since the body does not synthesize sterols, they must be ingested daily [37]. They can be found in many vegetable-based food sources, mainly in vegetable oils, nuts and cereals, but commercial products enriched in these bioactive compounds include seed oils like corn, soybean, rapeseed oil and yogurts, milk, spreads, and margarine [38,39]. Phytosterol application in food products as esters is primarily due to improving their solubility [37].

To date, phytosterols have been proven to reduce cholesterol absorption and lower plasma low-density lipoprotein [38,40]. Although, according to the World Health Organization, high cholesterol levels are considered as a risk factor for ischemic heart diseases [41], phytosterols, in addition to promoting heart health, by reducing the risk of cardiovascular diseases, also have strong anticancer [42] and antioxidant activities [40]. The most representatives are campesterol, stigmasterol, and β- sitosterol, the latter the most common plant sterol [39].

Interestingly, mainly brown seaweeds are enriched in fucosterol and saringosterol [7]. These bioactive compounds could permeate the brain-blood barrier and accumulate in the central nervous system, where they may, therefore, exert their neuroprotective effects [7].

The seaweed-derived sitosterol possesses neuroprotective potential through an anti-inflammatory effect and reducing Aβ plaque formation [7].

The phytosterol saringosterol also showed antineuroinflammatory functions reducing Aβ plaque formation, increasing Aβ clearance, and counteracting memory deficits, which can potentially overcome neurodegeneration [7].

The most common marine macroalgae sterol, fucosterol, possesses several biological properties, such as antioxidant, anticancer, antidiabetic, anti-inflammatory, anti-obesity, and regulating cholesterol levels [43]. Relatively to antioxidant activity, it increased the activities of some crucial antioxidant enzymes while decreasing the serum transaminase activities [44].

Moreover, fucosterol also inhibits cholinesterases, prevents the formation of Aβ peptides, among other effects suggesting that fucosterol can attenuate aging-associated cognitive decline [7,44].

These non-polar compounds are extracted, preferably using Soxhlet Extraction, among the conventional techniques from natural matrices. Various non-conventional techniques have been extensively used, and preferentially SFE. However, Soxhlet extraction is still considered a reference to newly developed methods [45].

### 2.5. Amino Acids, Bioactive Peptides and Proteins

Amino acids like histidine, leucine, isoleucine, valine, taurine and mycosporine are present in various seaweeds, exhibiting potential biological activity as antioxidants [6]. The most abundant amino acids were glutamic and aspartic acids in most species, while methionine content was lower in most of the species [46]. Bioactive peptides are low molecular weight protein fragments of 2 to 30 amino acid residues that display health-promoting effects after being released from their original protein during gastrointestinal digestion and through fermentation, enzymatic hydrolysis, and food processing [47,48]. These include antidiabetic [49], antihypertensive, antioxidant, antithrombotic, antimicrobial, and immunomodulatory properties [46].

The marine bioactive peptides biological activity depends on their chemical structure [46], composition, and amino acid sequence [49]. These differences could also be attributed to biotic and abiotic factors that affect the seaweed composition and structural modifications of the molecules during extraction and purification processes [46]. Products commercially available in Japan, containing seaweed-derived bioactive peptides such as a *U. pinnatifida*-derived peptide (YNKL), and a *Porphyra yezoensis* Ueda (=*Neopyropia yezoensis* (Ueda) L.-E.Yang & J.Brodie) derived peptide (AKYSL), claim to have antihypertensive properties [50]. Seaweed-derived bioactive peptides usually are obtained after protein extraction, fractionation, isolation, and concentration [50]. The obtained fractions can be used as substrates to prepare the bioactive peptides. Seaweeds phycobiliproteins and lectins are reported to be the most important proteins to supply peptides with various bioactive properties [50]. Enzymatic hydrolysis of *Porphyra dioica* J.Brodie & L.M.Irvine originated a protein hydrolysate with antioxidant, DPPIV and ACE inhibitory activities [51], while an *Ulva lactuca* L. hydrolysate presented ACE and renin inhibitory activities [52]. The bioactive properties of seaweeds derived-peptides and protein hydrolysates such as in vitro antioxidant, anti-inflammatory, anti-acetylcholinesterase, cardioprotective (renin inhibition, ACE inhibition), immunosuppressive, dipeptidyl peptidaseIV (DPP-IV) inhibitory, α-amylase inhibitory, tyrosinase inhibitory, in vitro antiproliferative, DNA damage protective effects, and antihypertensive effects have been recently reviewed in the literature [46,50,53,54].

Seaweed protein content differs from species to species, being generally lower in brown seaweeds than green or red seaweeds [6,46].

Moreover, different classes of proteins are considered good choices for generating biologically active peptides, emphasising lectins [46]. Lectins are carbohydrate-binding proteins that mediate bacteria, viruses, fungi, tumour cells, and erythrocytes attachment and binding [46]. In addition, they are known to play important roles in immunological applications and agricultural and therapeutic areas due to their antitumor, antiviral, and deleterious effect on microorganisms [55]. Lectins obtained from the green seaweed *Caulerpa cupressoides* (Vahl) C.Agardh, the red seaweeds *Pterocladiella capillacea* (S.G.Gmelin) Santelices & Hommersand, *Hypnea cervicornis* J.Agardh or *Solieria filiformis* (Kützing) Gabrielson [56] were reported to present anti-inflammatory effects in the paw edema model in mice. Lectins from the red seaweed *Bryothamnion triquetrum* have shown biological activities such as antinociceptive, vasorelaxant effects and anti-inflammatory activity [56]. Lectins from red seaweeds have also been recently connected with potential antiviral activity against influenza and herpes virus, HIV-1 in vitro, SARS-CoV-2 and other coronaviruses [57,58,59].

Besides, the antioxidant peptides usually present in animal muscle, carnosine and glutathione, have been found in some seaweed species [43], reinforcing the possibility of using seaweed-derived proteins to generate antioxidant hydrolysates and peptides.

Traditionally, aqueous and alkaline extraction, in addition to polysaccharide-aided extraction, are the usual methods used to extract macroalga proteins [6].

### 2.6. Omega-3 Fatty Acids

Omega-3 fatty acids are long-chain polyunsaturated fatty acids (PUFAs), also known as omega-3 or n-3 fatty acids, characterized by a double bond at the third carbon atom from the methyl end in their chemical structure [43]. They are essential constituents of animal lipid metabolism, playing an essential role in the human diet and physiology.

PUFAs are essential nutrients, which cannot, or only to a limited extent, be synthesized by mammals. Therefore, they must be obtained through diet [60]. The two main PUFA classes are omega-3 (n-3) and omega-6 (n-6), provided by fish or plant sources and vegetable oils, respectively [60].

Omega 3-fatty acids, mainly eicosapentaenoic and docosahexaenoic acids, present beneficial effects on dyslipidemia [43] and perform a role in diminishing cardiovascular risks [6]. Eicosapentaenoic and docosahexaenoic acids are mainly found in fish oil, which composition is mainly related to the fish diet consisting of marine algae and phytoplankton [60]. Fatty acids content in seaweeds is dependent on the harvest season, habitat and genetics [61]. Brown and red seaweeds usually contain higher amounts of PUFAs when compared with green macroalgae. This is because they present more beneficial omega-6/omega-3 and PUFA/saturated fatty acid ratios for human health [43]. Van Ginneken et al. [60] determined the PUFA content of several seaweeds (*U. lactuca*, *Chondrus crispus* Stackhouse, *Laminaria hyperborea* (Gunnerus) Foslie, *Fucus serratus* L., *U. pinnatifida*, *Palmaria palmata* (L.) F.Weber & D.Mohr, *A. nodosum, Caulerpa taxifolia* (M.Vahl) C.Agardh, *Sargassum natans* (L.) Gaillon). Palmitic acid (C16:0) was present in all species at relatively high amount. The lowest absolute value was measured in *C. crispus* (2.7 mg/g dw, 19% of total FA) and the highest absolute value in *F. serratus* (7.3 mg/g dw, 19% of total FA). The n-9 FAs were between 3% (*P. palmata*) and 56% (*A. nodosum*). The n-3 PUFA α-linolenic acid (C18:3) had the highest concentration in *U. lactuca* (4.5 mg/g dw, 20% of total FA) and the n-3 PUFA eicosapentaenoic acid (EPA, C20:5) was the most abundant FA in *P. palmata* (8.3 mg/g dw, 59% of total FA). Concerning the n6:n3 PUFA ratio, while *P. palmata* and *C. taxifolia* showed the lowest values (0.05 and 0.11), *A. nodosum* and *F. serratus* recorded the highest ones (2.75 and 2.44). Nonetheless, all the reported values are below 10, which is the threshold recommended by WHO for n-6: n-3 ratio to prevent inflammatory, cardiovascular and nervous system disorders [60,62].

Omega-3 fatty acids exhibit positive effects on the central nervous system for developing the brain, retinal, and neural tissues in fetuses and young children [6] and anti-inflammatory potential [43]. Therefore, it is of utmost importance to maintain the balance of n-3 and n-6 in the diet, given the beneficial effects on human health [60]. Omega-3 fatty acids exhibit anti-inflammatory and antioxidant activity, which may contribute to their beneficial cardiac effects and the prevention of breast cancer [43,60]. Conversely, primarily n-6 fatty acids are pro-inflammatory and tumour growth promotors [60].

Currently, it has become clear that eicosapentaenoic and docosahexaenoic acids are major components of brain cells and crucial for proper development and functioning of the brain and the nervous system, besides their action in preventing cardiovascular diseases [60].

Conventional extraction of fatty acids includes chloroform solvent extraction, but recently extraction technologies such as supercritical fluid extraction and ultrasound have been employed to extract these bioactive compounds [6].

### 2.7. Tocopherols

Tocopherols are fat-soluble compounds consisting of 8 different homologs with a chromanol ring with a phytol chain [63]. This group of fat-soluble compounds with activity similar to vitamin E, is widely known for its antioxidant activity [64].

**Figure 6:**
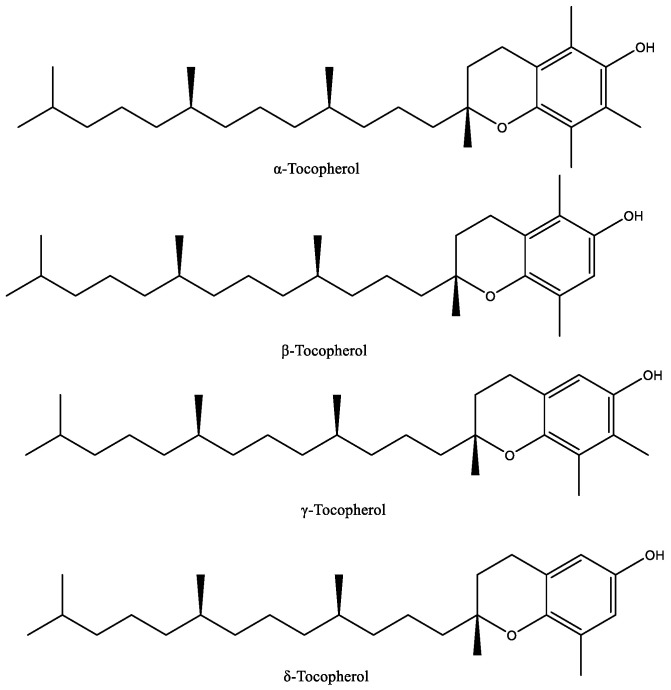
The four isomers of tocopherol: α-, β-, γ-, and δ-tocopherol.

Tocopherols have four isomers, α-, β-, γ-, and δ-tocopherol (Figure 6), that differ in the degrees of methylation on the chromanol ring, with α being trimethylated, β and γ being dimethylated in different positions and δ being monomethylated [63].

Tocopherol is synthesized only in photosynthetic organisms, which means animals obtained this antioxidant through diet [63]. Vegetable oils, including soybean, sunflower, almond oil, and peanuts, asparagus, tomatoes, and carrots, are rich in tocopherol [64]. Some animal fats also contain lower amounts of tocopherol [64], because as mentioned before, they ingest it from primary sources, like plants. This group of organic chemical compounds is frequently used by the food industry, owing to their efficient radical scavenging activity [22].

Brown algae present high contents of α-, β-, γ- and δ-tocopherol, while red and green algae contained only low levels of α-tocopherol. In general, brown algae were shown to contain higher levels of tocopherols than green and red algae [1].

The in vivo effect of different isomers of tocopherol and tocotrienol is debatable. According to Azzi [65] only RRR-α-tocopherol demonstrated beneficial effects in patients with inherited vitamin E deficiency, the so-called ataxia with vitamin E deficiency (AVED). In addition, some clinical trials have shown a significant reducing effect of α-tocopherol supplementation on serum concentrations of C-reactive protein and interleukin-6, highlighting its anti-inflammatory activity. In contrast, others did not find any significant effect, being the effect observed related to the vitamin E concentration tested [66]. On the other hand, Miyazawa et al. [67] referred that all vitamin E isomers exert in vivo effects, although to a different extent. All isomers are preferentially released into the bloodstream due to the presence of α-tocopherol transfer protein (α-TTP) in the liver, with the following relative affinities of 100% for α-tocopherol, 38% for β-tocopherol, 12% for α-tocotrienol, 11% for SRR-α-tocopherol, 9% for γ-tocopherol, 2% for δ-tocopherol, 2% for α-tocopherol acetate and 2% for α-tocopherol quinone. The vitamin E isomers unbound to α-TTP are then metabolized by phase I metabolism (catabolism and side-chain shortening), and phase II metabolism (sulfation and glucuronidation) and the resulting metabolites are then excreted from the body via feces and urine. The low affinity of some isomers for α-TTP is reflected in their different distribution patterns, with α-tocopherol being found in different organs while the other isomers, for example, α-tocotrienol, are tissue-specific. Indeed, it is thought that because of its poor affinity with α-TTP, tocotrienol is unlikely to be liberated from the liver into the bloodstream, but instead of via the lymphatic system reaching only some tissues. Taken together, and because of the extensive distribution of α-tocopherol compared with the other tocopherols and tocotrienols, current data seems to indicate that the antioxidant and anti-inflammatory effects of vitamin E are mainly attributed to α-tocopherol [67].

The extraction of these lipidic compounds is efficiently carried out using chloroform:methanol as a solvent mixture [22].

## 3. Seaweed Extracts: Purification Techniques

The consumption of algae is beneficial for health, but unprocessed marine algae can present potential health risks due to toxic elements [7]. Seaweeds may be contaminated by high iodine content and potentially toxic elements (Cd, Hg, and Pb), restraining the market expansion [1,4]. In addition, the regular consumption of wild seaweeds may lead to the risk of toxicity in humans, and high iodine status can impair thyroid function [68]. Consequently, the extraction of bioactive compounds instead of the direct use of algae is a way that may prevent excessive ingestion of heavy metals, which can be mutagenic and carcinogenic to humans [69].

The purification of the extracts isolating the bioactive or nutritional components from seaweeds may mitigate ingestion of excessive toxic components, ensuring healthy and safe products for commercialization [69,70].

**Figure 7:**
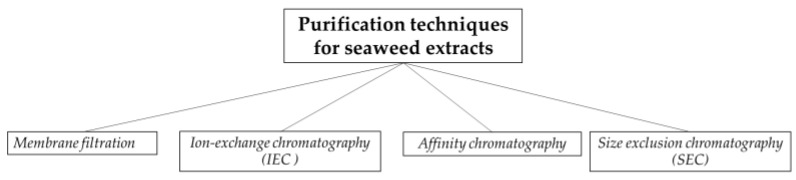
Examples of the most common purification techniques for seaweed extracts.

Purification techniques enhance and enrich the extracts with a mixture of compounds of interest with selective and desirable amounts [71]. The most frequent applied purification methods are membrane filtration and chromatographic techniques like ion-exchange, size exclusion, and affinity chromatography (Figure 7) [72].

### 3.1. Membrane Filtration

The membrane filtration principle is to purify molecular compounds based on the molecular weight of the target compounds [73]. This technique is based on the membrane’s selective permeability in allowing the substances of interest to pass through the membrane, while the unwanted compounds are generally retained in it [74]. Dialysis of the sample mixture placed in a membrane with ranging molecular weight cut off is a step described to effectively remove salts and contaminants from the extracts [71].

The use of membranes with ranging molecular weight cut off has been reported for purifying fucoidan and laminarin from brown seaweeds [71,73].

This technique presents several advantages that contribute to its extensive application in several fields: high efficiency, simple equipment, easy operation and low energy consumption [74], as well as the fact that it is suitable for scale industrial purification processes due to automated setup and ability to separate large volumes of solutions [73].

Membrane-based techniques’ greatest problem is membrane fouling, which leads to low performance and resolution, high energy inputs, and frequent replacement of membranes [71,73]. Zhu et al. [75] extracted pigments such as chlorophyls and carotenoids from *U. pinnatifida* using ultrasound-assisted extraction and recovered the pigments from the extract using 5 kDa and 10 kDa ultra-filtration membranes. One of the objectives was to study the membrane fouling mechanism and the threshold flux during filtration. These authors reported that the 10 kDa membrane was permeable to most pigments, while in the 5 kDa some pigments were partially retained. They also concluded that the cake layer was the most responsible for the fouling resistance during filtration [75].

Despite being an inevitable disadvantage of these techniques, fouling can potentially be overcome with tangential flow filtration, a combination of sequential ultrafiltration and diafiltration with decreased membranes with ranging molecular weight cut off [71].

### 3.2. Ion-Exchange Chromatography

Ion-exchange chromatography (IEC) is a broadly applied form of column chromatography used to separate charged molecules in a wideness of research, analysis, and industrial-scale purification processes [76].

The separating principle consists of the adsorption of charged molecules onto immobilized ion exchange groups of opposite charge and later elution of the sample by changing the pH or concentration of the running buffer [71,73].

Conjugating the high efficiency and resolution to the large sample handling capacity, cheap maintenance, and automation, IEC is the most frequently used chromatographic-liquid technique for purification purposes [71,73].

Polysaccharides, proteins, amino acids, and nucleotides are commonly purified using this technique [71]. For polysaccharide purification, anion-exchange chromatography is the most suitable method since fucoidan, the most available brown seaweed polysaccharide, exhibits high anionic charges due to sulfate ester groups linked into the carbohydrate backbone [73]. Ermakova et al. [77] isolated fucoidans from brown seaweeds *E. cava*, *Sargassum horneri* (Turner) C.Agardh, and *Costaria costata* (C.Agardh) De A.Saunders using ion-exchange chromatography. Anion exchange chromatography is commonly used to separate alginates and sulphated polysaccharides such as fucoidan and carrageenan [46]. On the other hand, because proteins have the particularity of possessing both negative and positive charges that varies according to the pH, the two ion-exchange chromatography types can be applied [76]. Phycoerythrins were isolated from aqueous extracts using a diethylaminoethyl column chromatography from *Ceramium isogonum* Harvey and *Polysiphonia urceolata* (Lightfoot ex Dillwyn) Greville, while a Q-Sepharose column chromatography was used to purity *Portieria hornemannii* extracts [78].

### 3.3. Affinity Chromatography

Affinity chromatography is based on the reversible interaction between the molecules and a specific ligand coupled to a chromatography matrix that presents specific affinity [73]. This interaction can occur between enzyme and substrate, antigen and antibody, receptors, and proteins due to ionic, hydrophobic, hydrogen and disulfide bond linkage, enabling the purification of these biological compounds [71].

In protein purification, immobilized dyes are used (dye-affinity chromatography) to specifically bind different proteins [73]. When applying this purification technique to polysaccharides like fucoidan, the process is based on the binding ability of fucose to lectin, resulting in a specific interaction that effectively purifies fucoidan from crude extracts [71]. This technique was used to isolate fucoidan from *Fucus vesiculosus* L. extracts [79]. However, the sulfate content of the polysaccharide can interrupt the binding of fucose subunits by interacting with lectin, affecting the efficiency of the purification process [73]. Fucoidan from brown seaweeds has been highly purified using a new dye-affinity chromatography method based on modified amino-derived sepabeads with toluidine blue [80].

### 3.4. Size Exclusion Chromatography

Size exclusion chromatography (SEC) is a partition chromatography that separates molecules according to their size, influenced by molecular weight and structure [81].

This method uses a physically, chemically stable, and inert porous matrix without interactions with the injected sample components [82]. Thus, as the sample travels through the column, molecules smaller than the pores can go through the pore of the matrix and be immobilized in the resin, while larger compounds cannot fit into the beads and are excluded from entering the pores [83]. For this reason, solutes varying in size will gradually become separated while travelling through the column, being the smaller ones eluted last from the column, and larger solutes will emerge first since they can only pass through the spaces between resin beads travelling a shorter distance overall [82,83].

SEC is a quick, reproducible, economical, and simple separation mechanism that preserves the desired compounds’ molecular structure and biological activity. Therefore, it is commonly applied to purify large macromolecular compounds like polysaccharides, proteins, and others [71]. For example, according to their molecular weight, SEC can be employed to purify laminarins and fucoidans of brown seaweed polysaccharides [73]. Furthermore, this technique helps to purify and desalt compounds with higher salt concentrations [71].

## 4. Identification, Characterization, and Quantification of the Bioactive Compounds from Seaweeds Extracts

The wide range of bioactive compounds present in the algae extracts creates the need for purification techniques to facilitate the target compounds’ identification, characterization, and quantification. However, the biochemical analysis of algae extracts is, in practice, more challenging compared to materials from terrestrial plants due to the lack of reliable, standardized analytical protocols for the analysis of algae molecules [84].

In this context, several chromatographic techniques coupled with different suitable detectors such as UV-vis detector, photodiode array, mass spectrometer and nuclear magnetic resonance (NMR) spectrometer as well as non-chromatographic Fourier-transform infrared spectroscopy (FTIR) [84,85] have been studied to determine and identify bioactive compounds.

Chromatographic techniques have been widely employed to identify several compounds from seaweeds. The most used and available in the research laboratories are HPLC with UV and DAD detectors, while MS detection might not be as widely available due to its highest cost. Although most HPLC separations are carried out using UV detection, due to its poor limit of detection (LOD), the resulting HPLC method can be unsuitable for determining the analytes present in small amounts in the sample [86]. With UV and DAD detectors, besides having a lower detection limit than MS detectors, the identification of the compounds in a sample is based on the comparison of retention time and spectra of the available standards [87]. More recently, HPLC coupled with MS or MS/MS detection allows for identifying and elucidating the compounds detected. The MS detector measures ions’ mass-to-charge ratio, allowing the ions to be sorted based on their mass. When using two mass spectrometers in MS/MS, molecules with a particular mass-to-charge ratio can be chosen to undergo further analysis by fragmenting the ion. The mass spectrum of the sample can be used to assess the concentration of compounds, find the mass of impurities and give insight into chemical structures. The highest limitation to this technique is the lack of standards (for phlorotannins, for instance) and reliable libraries for comparison [87]. When coupled to MS, HPLC is used to study high and low molecular weight compounds and their distributions in an extract. However, specialised equipment such as MALDI-ToF and HRMS is usually required to detect larger molecules such as proteins and phlorotannins. However, these types of equipment are usually not readily available and are very expensive [87].

HPLC coupled to NMR spectroscopy has also been used to identify structures of unknown complex molecules, with the advantage of being a non-destructive technique [84]. NMR is the most uniform detection method and allows for the unambiguous identification of compounds, although it presents relatively low sensitivity compared with MS [84]. However, NMR spectroscopy is the most effective method to elucidate the linkage position and isomeric forms of a compound. The ratio of linkages present in a complex matrix such as seaweed can be assessed using ^13^C-NMR spectral data, and besides, when using this technique, it is not necessary to perform a purification step [87]. In the future, linking the structures of compounds obtained by NMR spectroscopy to HPLC retention times and UV spectral data would be extremely useful for researchers.

### 4.1. Thin-Layer Chromatography

Thin-layer chromatography (TLC) is a relatively rapid, inexpensive, and straightforward process that allows the identification of the presence of a specific compound in a mixture when the Rf of an unknown compound is compared with the Rf of a known compound [85].

Identical molecules will invariably travel the equivalent distance under similar conditions. However, molecules that travel the same distance are not necessarily the same compound [88].

TLC is a highly sensitive method for analysing phytoconstituents compounds, whose application is limited due to the ease with which microorganisms grow on the surfaces of the TLC plate [85]. In addition, the complete removal of residual low volatile solvents and the transfer of the active compounds from the stationary phase into the agar layer by diffusion are problems that affect the efficiency of the method [85]. TLC coupled with appropriate detectors is an efficient analytical methodology that identifies and isolates phenolic compounds like phlorotannins [89] and sulfated polysaccharides [90]. TLC silica gel coated plates and the developing solvent (n-hexane: acetone) in a ratio of 7:3 were used for screening the presence of fucoxanthin in the extracts of five brown seaweeds: *Sargassum wightii* Greville in J.Agardh, *Sargassum ilicifolium* (Turner) C.Agardh, *Sargassum longifolium* (Turner) C.Agardh, *Padina* sp. and *Turbinaria* sp. [91].

### 4.2. High-Performance Liquid Chromatography

High-performance liquid chromatography *(HPLC)* is a modern, robust, and popularly used separation method, which is also used for identification, quantification and fingerprinting of compounds from a crude mixture [92].

LC-MS is an exact and robust method usually used to characterise peptide sequences, although it is time-consuming and high cost [93]. Therefore, these combinations allow rapid and accurate identification of chemical compounds, especially when a pure standard is unavailable [85]. LC-MS is used to quantify compounds such as flavonoids and tannins [94] and non-volatile molecules like betaines [95] in seaweeds.

HPLC-DAD was employed for the identification and estimation of β-carotene, fucoidan, dieckol [84], polyphenols [96,97], including phlorotannins [87], other carotenoids, tocopherols [94] and fucoxantin [98,99].

### 4.3. Gas Chromatography—Mass Spectrometry (GC-MS)

Gas chromatography coupled with mass spectrometry (GC-MS) is another hyphenated technique that allows the identification and quantification of a variety of volatile compounds and semi-volatile [100].

This technique combines the greatest separation power from chromatography with the specificity and sensitivity of a mass spectrometer, which can provide detailed structural information on most compounds, being a good approach for characterization and quantification of an organic analyte [101]. GC-MS separates different compounds in the sample based on their volatility by flowing an inert gaseous mobile phase, moving the sample through a stationary phase placed in the column [100]. Then, posteriorly, the compounds are identified and quantified by the mass spectrometer according to their mass-to-charge ratio (*m/z*) [100].

The major limitation of this technique is that the compounds must be sufficiently volatile to be transferred from the liquid phase to the mobile gas, thereby eluting from the analytical column to the detector [102]. For this reason, in some cases, volatile forms of the analytes must be produced by chemical derivatization [102]. However, unlike LC-MS, GC-MS allows an easy match with compounds in the National Institute of Standards and Technology database due to the stronger ionization energy that leads to many fragment ions [84]. GC-MS is used for the identification of sugars and lipophilic compounds like fatty acids and sterols [84], tocopherols, flavonoids and anthocyanins [94] and volatile organic compounds [103] from seaweeds.

### 4.4. Tandem Mass Spectrometry

Tandem mass spectrometry (MS/MS) is a technique for structural characterization and compound identification that uses two or more different types of mass analyzers arranged sequentially in tandem to enhance analysis through collision-induced dissociation [104]. MS-MS is a two-step method where firstly, the separation of a predetermined set of m/z ions from the ion source and consequent fragmentation by chemical reaction and secondly, mass spectra are produced for the fragments and used for structural characterization of the selected ion [101,105].

This technique is characterized by its high selectivity and specificity, low consumable cost, ability to measure very low concentrations of analytes, and ability to measure multiple analytes in a single method [102].

MS-MS is the leading technique for the determination of peptides and other biomolecules [106], is used to quantitatively and qualitatively study arsenosugars [84,107] and phlorotannins from seaweeds [84].

### 4.5. Fourier Transform Infrared Spectroscopy

Fourier-transform infrared spectroscopy *(FTIR)* is a powerful technique for identifying and characterising compounds in detecting functional groups present in a mixture of plant extracts [85]. This technique is based on the vibrations of atoms in a molecule [108], and it can be used for a wide range of materials as a qualitative or quantitative analysis [109]. The peaks in the infrared spectrum represent the excitation of vibrational modes of the molecules and thus are associated with the chemical bonds and functional groups. In contrast, the absorbance of a molecular vibration represents the amount of infrared energy absorbed by a compound, and it is proportional to its concentration [110]. FTIR device has largely replaced the old dual beam unit due to their better spectral resolution, faster data collection and improved signal-to-noise ratio [111].

This method is a well-established tool for protein structural characterization [112], is also used to characterize polysaccharides from marine algae-like fucoidan, carrageenan and alginic acid [113] and phenolic compounds [114].

## 5. Applications of Seaweeds and Their Extracts for Human Use

For the applications of seaweeds and their extract for human use, first is necessary to assess potential contamination and toxicity problems. Despite that, working with natural compounds presents some other problems, such as stability and bioavailability problems, chemical degradation reactions during storage, and sensitivity to oxidation and photo-oxidation, that need to be overcome, for example, with nanoencapsulation techniques [115]. Encapsulation can be achieved by encasing bioactive compounds in solid, liquid, or gaseous states in matrices, released under a controlled rate [116]. Nanocarrier systems can be valuable ways to improve the delivery of biologically active compounds [115]. Many of these compounds are poorly soluble in aqueous systems, which is another problem that can be solved using nanoencapsulation [116]. The mixture of the extract itself or the nanoencapsulated ones, with different matrices, facilitates its application in several industries [116].

Fucoxanthin is a major carotenoid pigment that occurs in the chloroplasts of brown seaweeds. Although the exact benefits in human health of this natural molecule, when used as a food constituent, is not determined, fucoxanthin has been studied clinically for its antioxidant properties, anticancer, antidiabetic, antiobesity, anticholesterol, anti-inflammatory, antiangiogenic, antimalarial, and antihypertensive activities [117]. However, the characteristic insolubility, pH instability, sensitivity to oxidation, and impaired bioavailability of fucoxanthin limit its potential beneficial actions when incorporated in food products [117]. This obstacle can be overcome with several approaches that include microemulsions and nanoencapsulation. The use of microemulsions composed of a water phase, lipid phase, and an amphiphilic compound to encapsulate fucoxanthin appears to increase its suitability when incorporated into the hydrophilic matrix for food and pharmaceutical applications [118]. In addition, studies demonstrated an oil-in-water microemulsion’s efficiency for the hydrophobic fucoxanthin delivery to aqueous food systems [117].

In another study, an increase in β-carotene bioaccessibility was related to decreasing droplet size from small to large in an emulsion-based delivery system using corn oil-in-water emulsions with different initial droplet diameters: large [119]. Polysaccharides can be used in food emulsions to deliver oil-soluble flavor substances, functional oils, bioactive peptides, polyphenols, carotenoids and probiotics, delaying lipid digestion, prolonging satiety and improving target delivery [120]. Polysaccharides show better resistance to the enzymatic and gastric acid environment. The environmental conditions (heat, pH, light, oxygen) and physiological digestive conditions (pH, enzymes, intestinal barrier) are responsible for the low absorption and bioavailability of bioactive components from foods [120]. The emulsion effectiveness as a delivery system is that it protects the encapsulated components until the targeted location is reached. It was reported that polysaccharides, such as chitosan, pectin, carrageenan, starch, alginate, and methylcellulose, have a potential effect on the gastrointestinal fate of food emulsions [120]. In an in vitro study simulating the human digestion, oil in water emulsions stabilized with polysaccharides from *Ulva fasciata* Delile loaded with β-carotene, the oil droplet size increased after mouth-stomach digestion stage. After the intestinal digestion stage, the size decreased attributed to the catalysis by cholate and pancreatic enzymes [120]. Polysaccharides as emulsifiers have the advantage that the produced oil droplets are relatively stable to changes in ionic strength, pH, or temperature because they can act as thickeners to stabilize the emulsion and potentially influence the gastrointestinal fate of the encapsulated bioactive food components. Polysaccharides alone can exhibit emulsification ability, but they usually interact with other emulsifiers to synergistically improve the stability of emulsions [120]. Polysaccharide–polysaccharide, polysaccharide–surfactant or polysaccharide–protein interaction can produce stable oil in water emulsions improving nutrient absorption being this a potential advantage to the use of seaweeds as food, due to its richness in complex polysaccharides and other components.

Additionally, encapsulation with nanogels was reported to significantly improve fucoxanthin’s biological availability and stability [121]. Besides protecting the molecule from degradation, encapsulation can also prevent reaction with other ingredients and seaweed flavour or brown colour from immersing in food [122]. Still, when present under the different conditions found in the human body, the instability of carotenoids makes it difficult for these bioactive molecules to maintain their health benefits [123]. For this reason, nanoencapsulation technology, besides protecting these molecules from severe conditions and potential hydrolysis and oxidation, also increases the efficiency and bioavailability of the bioactive nutraceutical ingredients because of their small size, easy penetration into the cells and cell organelles, large surface area, and long-term stability [124,125]. Furthermore, apart from carotenoids, carbohydrate content in seaweed is considerably high, especially as polysaccharide fibres [126]. Therefore, these compounds present potential activities against cancer and virus, although their efficiency is low since the human body cannot take them due to their large size and irregular shape [126].

### 5.1. Seaweeds as Human Food

For centuries, seaweeds have been traditionally used as a food source in many Asian countries [5,127]. In contrast to this continent, their exploitation in Europe has been minimal and mainly focused on the industrial production of gelling agents [1].

Nowadays, the consumption of seaweeds increased considerably in many more countries outside Asia [5,26]. As a result, these marine functional foods have been produced and marketed, offering health benefits and the potential to reduce the risk of diseases [26].

Seaweeds have excellent dietary content, revealing relatively high protein levels, essential amino acids, carbohydrates, lipids, polyphenols, pigments, and vitamins [1,127]. Among these properties, ranges in shape, colour, texture, and taste make seaweeds appealing as food [1]. They are also one of the richest sources of natural antioxidants and antimicrobials [26], making macroalgae and their bioactive compounds food products more functional as health-improving against different diseases and nutritionally appealing.

Marine algae can be served in its dried form, powered, or extracts added to different food products such as meat, dairy, fish, or vegetable-based products, and it may play a significant role in health status if consumed throughout life as part of the daily diet [26,127]. Some typical food applications of seaweeds are presented in Table 2. However, it should be considered that the consumption of algae, especially in their whole form, can represent harm to consumers by cause of possible toxicity from high iodine levels in seaweeds, accumulation of arsenic and toxic elements, secondary metabolites as well as the presence of pathogens and radioisotopes [128].

The use of seaweeds extracts in the food industry is one of the most interesting applications to improve nutritional properties [117].

*A. nodosum* and *F. vesiculosus* Linnaeus extracts have been incorporated as components of yoghurts and milk, evidencing antioxidant functionality without compromising their shelf-life characteristics and quality parameters [26,134].

Likewise, fucoidan and laminarin polysaccharides isolated from a brown seaweed applied directly to minced pork, promoting preservation against food spoilage and pathogenic microorganisms, and decreased lipid oxidation in the cooked patties a viable alternative to reduce the use of chemical preservatives [134].

Additionally, dietary fibre composed of polysaccharides such as alginates, cellulose, fucans, fucoidan and laminarins extracted from *F. vesiculosus* was added to horse mince, promoting a potential antioxidant effect. This activity is due to the polysaccharides mentioned above and the presence of phenolic compounds (phloroglucinol and phlorotannins), vitamin E, and carotenoids [117,134]. The phlorotannins present in *F. vesiculosus* were also used to increase the antioxidant activity of fish products [133].

Macroalgae are also a source of extractable lipids, including PUFAs that may reveal future applications as dietary products for their anti-inflammatory properties on obesity and, consequently, in obesity-associated disorders [69].

The addition of seaweed extracts has been evaluated in addition to its application in meat products (chicken and pork), in seafood, bakery, and dough products like bread, as an opportunity to improve their nutritional and dietary goals as well textural and organoleptic properties [26].

Despite their advantages, a considerable difficulty of incorporating seaweed extracts in food products is the strong sensory impact on these functional products, which makes their commercialization challenging [134]. However, added to their potential applications as functional ingredients to increase the nutritional properties of food products, they can be used to improve the textural and consistency properties of food products [26].

Algae are characterized by their high content of phycocolloids that varies among different algal species from various taxonomic groups [25]. For instance, brown seaweeds contain many alginates, while red algae contain agar and carrageenan [25,127]. These polysaccharides are specially used as solidifying agents and gel-forming dietary supplements [5,25].

About 90% of the extracted agar is applied in food products due to its ability to form a gel with unique properties [24,25]. In this context, agar is used in the food production process of various products like ice cream, meringue, fruit puddings, jams, juice, candies, chocolates, and coffee [5,25,127]. In meat and fish products, it is used as a gelling agent, in dairy products as a texture improver, in baked products such as cakes and bread, it is used as a stabilizer and thickener [24]. Still, it can be used as a laxative, an appetite suppressant, as fat replacers, cryoprotectants that preserve food during the freezing/thawing process, and as edible films [23,26].

Many of these food applications take advantage of some properties of this polysaccharide, such as the temperature at which it melts that is unusually high (85 °C) and unique to agar when compared to gelatin gels (37 °C), which means in food applications, there is no need to keep them refrigerated in hot climates [5]. In addition, the mouthfeel is different from gelatin since they do not melt or dissolve in the mouth [5]. At the same time, agar has no taste and smell, so it does not interfere with the sensory characteristics of food [25]. 

Carrageenans are the most produced gelling hydrocolloids derived from classes of red algae like Rhodophyceae, Gigartinaceae, Hypneaceae, Solieriaceae, and Phyllophoraceae [23,25]. Since carrageenans are non-toxic, biodegradable, and biocompatible, increased interest in their abilities as gelling agents, stabilizers, emulsifiers, and thickeners in the field of the food industry has been verified [25,26,27]. Furthermore, this polysaccharide can bind water and improve palatability and appearance through interaction with other substances in the food [23]. For this reason, they are commonly applied as a dietary supplement in dairy products such as ice cream, yogurt, cheese, salad dressings, foams, and milk-based products to improve texture, thickness, and solubility, acting also as a stabilizer [23,26]. Likewise, carrageenans are successfully used to control discolouration, maintain texture, increase shelf-life, and preserve products such as sliced lychee, bananas, mangoes, and fresh meat and fish, by providing antibacterial protection coating [23,25,26]. Although, it should be noted that, contrary to agar, it cannot be digested in the human gastrointestinal tract [25].

Alginates are another class of algal polysaccharide commonly used in the food industry as thickeners, stabilizers, and emulsifiers [23,25]. Due to presenting properties like high biocompatibility, non-toxicity, ability to retain water to form gels when mixed with Ca ions, ability to increase the viscosity of aqueous solutions and ability to form films of Na or Ca alginate and fibres of Ca alginates, about 30% of produced alginates every year are intended for use in the food industry [25,27]. Another particularity of alginates is that, contrary to agar, they do not melt at high temperatures and form cross-linked gels and the fact they can maintain their properties for a long time at room temperature [25,26].

Alginate is mainly used as a potential ingredient and stabilizer in frozen foods and ice creams and in reduced-fat products, where it stabilizes the mixtures and provides higher viscosity, longer melting time, and better organoleptic properties [26,127]. In addition, it acts as an emulsifier for many food products, being comprehensively used as additives in instant drinks to keep food particles liquid in the mixture [27,127]. Alginate gels are also used in several restructured food products, such as meats for human consumption (chicken nuggets, roasts, meatloaves, and even steaks) and vegetable products and baked products [5,26].

Calcium alginate films and coatings have been used to help preserve frozen fish products by retarding the decay and improving shelf-life, improving the heat distribution and thus shortening the cooking time of chicken nuggets, protecting the meat from bacterial contamination and improving the sensory quality and reducing water-loss in a variety of foods [5,26]. Besides, alginate can also be applied as a carrier for antibrowning agents like citric and ascorbic acids, which preserve the colour of fresh fruits, with the added advantage of improving their antioxidant potential [26]. Furthermore, it has been reported that alginate can regulate appetite and, therefore, could be used as a supplement [26].

### 5.2. Seaweeds as Animal Feed

Seaweeds have been used as a part of the livestock diets since ancestral times and can potentially improve animal production performance and health [135,136]. In addition, they can build resistance to disease by guaranteeing a balanced micronutrients intake [127]. For instance, mastitis and cow fever may be decreased by the consumption of algae [127]. Some applications of seaweeds as animal feed are presented in Table 3.

*A. nodosum* has been the primary raw material for seaweed meals for decades. It contains essential amounts of elements (K, Na, and Cl), sulfated polysaccharides (fucoidan), and vitamins [136,141] that are the main contribution to the nutritional value of seaweed in the diet of animals. They also improve the fat level, iodine content, yield milk, and enhance animals’ fertility and birth rate and improve yolk colour in eggs [127]. The use of *Sargassum dentifolium* (Turner) C.Agardh to feed laying hens, shows to decrease the cholesterol content in the yolk, and to increase fatty acids, triglycerides and carotenoid content in eggs and in the plasma [140]. Furthermore, studies demonstrate that *A. nodosum* meal and its extracts can enhance immunity and antioxidative status in cattle, sheep, and goats by increasing superoxide dismutase activity [142]. Also, these algae extracts contain significant concentrations of phlorotannins that play a role in reducing ruminant fermentation. The introduction of *A. nodosum* extracts in the feeding of steers promoted a decrease in body temperature associated with fever caused by fescue toxicosis [143]. Furthermore, the presence of sodium chloride and potassium gluconate in *A. nodosum* extracts is revealed to reinforce immunity, improve health status, and protect against prolonged heat or transport-induced oxidative stress of the animals [141].

Tasco is an animal feed product manufactured with *A. nodosum* and its extracts, which presents the particularity of retaining all the bioactive and small molecules of this macroalga that play a predominant role in its efficiency when applied in animal rations [135]. It contains representative amounts of diverse, complex carbohydrates and polysaccharides such as alginic acid, fucoidans, mannitol, and laminarin [135]. Beneficial effects include resistance to stressors [142], increased competency of the immune system [144], increased productivity and/or quality of the animal [137], and a marketed reduction of pathogenic microorganisms that may cause foodborne diseases [138].

Some algae like *Gracilaria* sp., *Gelidiella* sp., *Hypnea* sp., and *Sargassum* sp. are also used to feed fish cultures [127]. Because of that, the enriched feed with minerals, amino acids, and carbohydrates promotes the maintenance of water quality in aquaculture [127]. Besides, seaweeds can be used as a water disinfectant in aquaculture [127]. There is also a market for fresh seaweed rich in protein as a feed for abalone, showing that growth is greatly improved [5].

Besides the nutritional feeding properties, seaweeds derived compounds like alginate is used as a binder that holds together food, preventing it from disintegrating or dissolving in the water [5].

### 5.3. Other Human Applications of Seaweeds

#### 5.3.1. Seaweeds as Pharmaceutics and Medicinal Products

Macroalgal constituents are about 35% in newly discovered chemicals for pharmacological and medicinal uses [145]. For centuries, seaweeds have been widely used as the origin of effective nutritional supplements [127]. Apart from this support, they are also used in therapeutics as antimicrobial, anti-viral, anti-fungal, antiallergic, anticoagulant, anticancer, anti-fouling, antioxidant and neuroprotective activities [7,127,145,146]. Table 4 presents the main bioactivities of some seaweed species with potential and interesting applications for the pharmaceutical and cosmetic industries.

The most notable algae species that showed anti-viral activities towards human infectious diseases such as HIV, HSV type 1 and 2 and RSV, are *Aghardhiella tenera* (J.Agardh) F.Schmitz and *Nothogenia fastigiata* (Bory) P.G.Parkinson, mainly due to the presence of sulfated polysaccharides [127,145,147,148,149]. In addition, other compounds like carrageenans, fucoidans, and sulfated rhamnogalactans have been reported to present anti-viral properties by inhibiting the binding of the viral particle to the host cell. However, other algal fractions have virucidal and inhibitory enzyme effects or slow down syncytium formation [127,145,147,148,149]. Therefore, an anti-viral polysaccharide must have very low cytotoxic activities towards mammalian cells, particularly polysaccharides of *A. tenera* and *N. fastigiata*, which have this characteristic [145].

Macroalgae contain several compounds responsible for their antibiotic properties [145]. These interesting substances include fatty acids, bromophenols, tannins, phloroglucinol, terpenoids, halogenated compounds such as haloforms, halogenated alkanes, alkenes, alcohols, aldehydes, hydroquinones, and ketones [127,145].

As seaweeds are very important reservoirs of new therapeutic compounds, some extracts have also proved to be potential protective agents against cancer [127,163]. The mechanisms in which cancer could be reduced or retarded include reducing cholesterol, binding of biliary steroids, antioxygenic and toxic materials activity, induction of apoptosis, inhibition of cell adhesion, and addition of important trace minerals to the diet [127]. Several sulfated macroalgal polysaccharides like fucoidans are known to have anti-tumour, anti-cancer, antimetastatic, and fibrinolytic properties, along with their capacity to reduce cell proliferation [145]. The brown algae *Fucus* sp. is active against both colorectal and breast cancers [127]. Also, chondriamide A shows cytotoxicity against human nasopharyngeal and colorectal cancer cells [145].

Moreover, algae are an excellent source of iodine that helps overcome Goitre disease, a consequence of low iodine intake [127,170]. Vitamin deficits can also be prevented using seaweed supplements in the diet [127].

Further, agar has been used for many years as a laxative, and alginic acid helps relieve heartburn and acid indigestion [5].

Apart from bioactive activities, these compounds derived from macroalgae are applied in the pharmaceutical industry and have functional ingredients. For example, sodium alginate solutions are used in wound dressings due to their excellent wound healing and hemostatic properties and the fact they can be absorbed by body fluids because the Ca in the fibre is exchanged for Na from the body fluid, which also makes these dressings not adhere to the wound [5]. Alginate is also used to deliver various drugs in a more controlled and slowly way [5].

#### 5.3.2. Seaweeds as Cosmetics

The cosmetic industry is persistently searching for new active compounds from natural sources, which are more environmentally friendly, present fewer side effects, and have a safer use [171]. For this reason, seaweeds are widely used on cosmetic products, particularly in the face, hand and body creams or lotions, but usually because of the properties mentioned above of alginate or carrageenan (gel, thickener, and emulsifier) [5].

However, macroalgae can be attractive to the cosmetics industry for the hydrocolloids and other reasons, namely the presence of bioactive compounds (phenolic compounds, terpenoids and sulfur, and nitrogen derivatives), minerals, polysaccharides, proteins, and lipids they can produce [172]. Some of these bioactivities important for the cosmetic industry are presented in Table 4.

Many formulations use algae extracts, mainly from *Fucus* sp. or *Laminaria* sp., with applications of interest for slimming and cellulite reduction purposes [170,172]. Their exceptionally high iodine content justifies their presence in these products since iodine is involved in thyroid metabolism. It is known that thyroid hormones promote lipolysis by increasing the penetration of fatty acids in the mitochondria due to the increased synthesis of carnitine palmitoyltransferase [172]. Even though the iodine amount depends on the alga species and the harvest location, it should be noted that algae have the property of concentrating the iodine from seawater. Since it is prohibited to use iodine in cosmetics, its replacement by seaweeds is an advantage [172].

Some tested formulations containing aqueous extracts from different macroalgae evidenced the ability to eliminate fats, synthesize pro-collagen I, and improve lipolysis-related mechanisms, culminating in the final gold of reducing cellulite [170,172]. For cellulite cases, compounds extracted from algae can stimulate tissue metabolism and blood circulation in the application area, thereby helping to mobilize the fat accumulated in the subcutaneous tissue [170]. Additionally, the body weight gain through the gene regulation and protein expression involved in lipolysis and lipogenesis is significantly reduced, highlighting the capacity of active ingredients from seaweeds to promote a weight-loss effect [170].

Ageing is a normal process associated with oxidation causing changes in the skin, like dryness, irregular texture, wrinkles, reduced elasticity, and volume, due to multiple intrinsic, genetically programmed, and extrinsic factors, such as UV radiation and toxins [170]. Some seaweeds attracted attention in the cosmetics industry because of their ability to nourish and rehydrate the skin, being the algae frequently used in cosmetics *Laminaria* sp., *Fucus* sp. (brown algae), and *C. crispus* (red alga) [170]. Several compounds extracted from seaweeds like vitamins B, C, D, and E are considered valuable in various skincare applications [171]. Algae are also rich in amino acids, especially serine, which are of particular interest here, as are those extracts rich in PUFA [172]. Fatty acids enable the reconstruction of the intercellular cement and thus reinforce the skin barrier, being linoleic acid particularly effective in this field among all fatty acids [172]. This fatty acid has also acted as an emollient that protects the skin from drying [171]. Products like seaweed paste mixtures are used in massage creams, with the potential to restore elasticity and suppleness to the skin [5].

Besides, some secondary metabolites of seaweeds have been demonstrated to be skin protectors by exhibiting activity to reduce ROS caused by UV radiation, preventing wrinkles, and delaying skin ageing [170,171]. Moreover, the topical application of fucoidan has been reported to possess anti-ageing properties by increasing cells hydration and elasticity [170]. Furthermore, molecules like eckol and dieckol have already proven to be metalloproteinase inhibitors [172]. Metalloproteinases are responsible for wrinkle formation in the skin [172], the reason why its inhibition is of extreme interest. Also, some carotenoids from seaweeds, such as astaxanthin with exceptional antioxidant properties much more significant than α-tocopherol, can be applied in the field of anti-ageing cosmetics [172].

Hair loss is a frequent problem with an increasing impact nowadays, related to different causes, including ageing, induced pathologies, or chemically promoted [170]. In this sense, many studies have focused on finding compounds capable of preventing or delaying hair loss and stimulating its growth [170]. For example, extracts of *E. cava* were reported to enhance the proliferation activity of hair follicles cells and to promote hair-shaft growth [173]. The hair-growth-promoting activity is likely derived from the dioxinodehydroeckol, a phlorotannin identified in *E. cava* [173]. Moreover, dieckol and 7-phloroeckol, also isolated from *E. cava*, were proven to cause capillary growth-inducing dermal papilla cell proliferation and inhibit 5 α-reductase enzyme activity [170].

Skin lightening products [172] are usually formulated using tyrosine inhibitors such as kojic acid or arbutin, being the first one suspected of possessing mutagenic activity and the second one to cause toxicological problems [172]. For this reason, the search for natural, non-toxic, and active skin whitening ingredients as tyrosinase inhibitors from seaweeds is of great interest [171]. Melanin, the pigment responsible for skin colour, is synthesized by tyrosinase. Sun exposure induces abnormal melanin synthesis, resulting in skin pigmentation [171]. Seaweed extracts of *Endarachne binghamiae* J.Agardh, *Schizymenia dubyi* (Chauvin ex Duby) J. Agardh, *E. cava*, *Sargassum siliquastrum* (Mertens ex Turner) C.Agardh and *Ecklonia stolonifera* Okamura have already shown potential for tyrosinase inhibition activity [171,172]. The carotenoid fucoxanthin exhibits tyrosinase inhibition activity when the subject is treated orally or topically, being this effect related to its antioxidant activity [171,172]. Likewise, the phlorotannin dieckol and the polysaccharide fucoidan demonstrated a promising tyrosinase inhibitor effect [171]. To summarize, the whitening properties of the derived algae cosmetics are based on their capability to inhibit melanin synthesis, decrease tyrosinase activity, control melanogenesis, and protect the dermal matrix against proteases, free radicals, and UVA/UVB radiations [172].

Wound healing is a regeneration process of the damaged tissues to fill the wound gap followed by dermal and epidermal cells proliferation and migration and matrix synthesis [174]. However, wound healing drugs are mostly unavailable, pricey, and present several adverse effects [175]. In this context, the search for potential wound healing compounds from diverse natural sources like seaweeds has been widely explored, not only because they are safe, medically effective, low cost, and good tolerance by patients, but mainly because of all bioactive substances compounds they produce [176]. Evidence suggests that bioactive compounds extracted from *S. ilicifolium* stimulate accelerated wound healing by promoting cellular proliferation and migration of fibroblasts [174]. Several other reports affirm the ability of phlorotannins like dieckol and eckol to repair skin damages from various allergens by attenuating the expression of MMP-1 (interstitial collagenase behind the degradation of dermal collagen in skin ageing process) in human dermal fibroblasts [170]. Fucoidans also present interesting potential effects in the growth of fibroblasts and epithelial cells, accelerating wound healing and modulating the growth factor-dependent pathways in tissue repair [170]. Therefore, fucoidan is employed in hydrogel cosmetics to enhance skin burns lesions effectively [177]. Along with this polysaccharide, carrageenans were also reported to improve significantly wound healing and hair growth [178].

#### 5.3.3. Other Applications of Seaweeds

Seaweeds have a broad range of applications, including crop fertilization, fuel production and wastewater treatment [127]. Table 5 shows some of these applications and the seaweeds used for these porpuses.

##### Seaweeds as Fertilizers

Seaweeds are used as general manure by coastal people throughout the world, especially the large brown seaweeds [5].

In this context, seaweed and seaweed extracts are rich in compounds that may be beneficial to plant growth and development, such as macro and micronutrients, trace elements (Fe, Cu, Zn, Co, Mo, Mn, Ni), vitamins, amino acids, and phytohormones (auxin, cytokinin, gibberellins, phenylacetic acid), that makes macroalgae an excellent fertilizer [127,179,180]. Furthermore, benefic effects after using these natural extracts on the growth and yield of plants, seed germination, tolerance to environmental stress, resistance to fungal disease and insect pests, enhanced antioxidant properties, and increased nutrient uptake from soil have been reported [181,182].

A notable advantage of using seaweeds like species of *Laminaria*, *Ascophyllum*, and *Sargassum* genera as manure is their biodegradability, non-toxicity, non-polluting, and non-hazardous nature to living beings [127]. In addition, alginic acid can be an interesting soil conditioner, and like laminarin, could reduce the severity of soil-borne fungal diseases considerably [181].

Whapham et al. [183] applied an alkaline extract from *A. nodosum* to the soil or the foliage of tomato plants, leading to the production of leaves with higher chlorophyll levels than control plants. The chlorophyll content was increased probably because of a reduction in chlorophyll degradation, which might be caused partly by betaines in the seaweed extract [183]. Moreover, during storage conditions, glycine betaine delays the loss of photosynthesis activity by inhibiting chlorophyll degradation in isolated chloroplasts [184].

In general, the presence of Mg and Fe in seaweed extracts revealed a potential influence in chlorophyll synthesis in plant metabolism by enhancing this photosynthetic pigment [184].

##### Seaweeds as Biomass for Fuel

The use of the unexploited biomass of macroalgae to produce biogas is a practice in most developed countries [127]. The most common algae for this purpose is *Macrocystis pyrifera* (Linnaeus) C.Agardh, because of its high growth rate and facility of mechanical harvesting [5]. Although other species of the genera *Laminaria*, *Gracilaria,* and *Sargassum* are also being investigated to determine their ability to be converted to methane by anaerobic fermentation [5]. Thus, it was verified that *Sargassum* sp. gave a small gas yield, especially when compared to *M. pyrifera* that presents good gas yields. However, dependent on the mannitol and alginate contents [5]. The relationship between these compounds and the methane yields is that the greater the content of mannitol more gas is produced [5]. On the opposite hand, for *Gracilaria* sp., the methane yield is closely related to the carbohydrate content and sometimes the protein content likewise [5].

##### Seaweeds as Wastewater Treatment

Seaweeds are explored mainly for their potential use to remove toxic elements from industrial wastewater and reduce nitrogen, phosphorus-containing compounds, sewage and some agricultural wastes before releasing these treated waters into rivers or oceans [5].

Seaweeds can take up high nitrogen concentrations and store more phosphorus than they require for maximum growth in their tissue, acting as a biofilter between wastewater and the pollutants [5,185]. Intertidal and estuarine species are the most tolerant, especially green seaweeds from *Enteromorpha* and *Monostroma* genera [127].

Concerning heavy metals, seaweeds can also reduce or remove their content from wastewaters [185]. However, heavy metals pose a danger to both the environment and living organisms, as they are toxic and carcinogenic, even at residual concentrations, with the particularity of being non-biodegradable and quickly accumulate in living organisms [185].

Cu, Ni, Pb, Zn, Cd, Cr, As, and Hg are the main alarming potentially toxic elements derived from industrial wastewater [127]. The absorption process as a treatment method for toxic elements decontamination appears to be more economical, practical, simple, and versatile when compared with others [185]. In this perspective, seaweed meets these criteria as it can absorb these toxic compounds to varying extents depending on the seaweed type and metal ion concentration [5]. The different cell wall composition between the red, green, and brown algae is the main reason for the variance in their affinity for metal biosorption [185]. Brown seaweeds such as *Sargassum* sp., *Laminaria* sp. and *Ecklonia* sp., and the green *Ulva* sp. and *Enteromorpha* sp. have been more efficient in accumulating toxic metals [127].

New approaches using algae extracts, instead of the commonly used whole seaweed or seaweed powders as adsorbents, were tested for the treatment of wastewater [186]. In general, the extracts effectively and drastically reduced compounds, including total dissolved solids, hardness, chloride, sulfate, and chromium from the effluent. Thus, this method for treating effluent appears to be an effective and economical treatment that may reduce harmful compounds present in polluted waters, with a potential application comparable to other techniques in the market [186].

## 6. Conclusions and Future Trends

Seaweeds are a valuable unexplored source of bioactive compounds that include vitamins, phenols, polysaccharides, proteins, carotenoids, and lipids, with a broad range of biological activities, ranging from anti-tumour, antimicrobial, and anti-ageing to anti-inflammatory, antioxidant and neuroprotective activity.

The incorporation of seaweeds or isolates of seaweeds, especially in the food, cosmetic, and pharmaceutical industries, reveals a promising potential for developing functional products, which can have a beneficial influence on human health. Although we have discussed the potential application of the main classes of compounds present in seaweeds, we cannot forget that, when the raw seaweed or a seaweed extract is incorporated in a food, pharmaceutical, or cosmetic product, the observed beneficial effect may be due to the synergistic interaction of different compounds.

Regarding the field of the food industry, the incorporation of algae extracts into the food systems demonstrates not only to improve the textural characteristics due to the action, especially of the hydrocolloids characteristic of these marine species, but also the organoleptic, nutritional and health characteristics of the final products. However, the sensorial impact these can bring to the final product is one of the most challenging problems for the commercialization of algae-based food products. It should also be noted that the effects of these products depend on the species of algae used since their constitution in bioactive compounds varies from species to species and the concentration used in the formulation of these products.

Nevertheless, seaweed consumption must be associated with an awareness of its potential risks to human health due to the possible presence of toxic contaminants such as heavy metals and their excessive iodine content. For this reason, the application of green extraction and purification processes of compounds from the complex seaweed matrix is a valid and logical strategy to avoid these health-related issues and to create added-value functional products due to the presence of a large variety of novel bioactive components with potential activities against several human diseases.

## Figures and Tables

**Table 1:** Proximate composition and total phenolic, total carotenoid, total tocopherol and total phytosterol contents of Seaweeds.

Class of Compounds	Green Seaweeds	Red Seaweeds	Brown Seaweeds	References
Polysaccharides (%, dw)	29.8–65	18–74	12.2–68	[11,12,13,14]
Proteins (%, dw)	4–44	6–50	1–24	[11,12,13,14,15]
Total lipids (%, dw)	0.2–4.1	0.12–3.8	0.3–4.5	[11,14,15]
Saturated fatty acids (% of total fatty acids)	23.5–77	25.5–85.7	15.8–50	[11,14]
Mono-unsaturated fatty acids (% of total fatty acids)	12.2–38.8	1.0–35.7	10.1–36	[11,14]
Poly-unsaturated fatty acids (% of total fatty acid)	6.6–39	9.1–68	17.8–70.9	[11,14]
Phenolic compounds (TPC, mg GAE/g dried extract)	1.26–50.0	1.05–38.08	0.26–397.23	[16]
Carotenoids (µg/g dw)	1.41–298.87	0.29–202.91	3.40–7.51	[17,18]
Phytosterols (µg/g dw)	1700–2100	186–337	662–2443	[19,20,21]
Tocopherols (µg/g dw)	19.70–35	14.25–500	3.63–450	[14,15]

dw—dry weight.

**Table 2:** Food applications of seaweeds compounds.

Seaweed Species	Compounds	Applications	References
*Gelidium* sp, *Gracilaria* sp., *Pterocladia* sp., *Gelidelia* sp.	Agar	Thickening and gelling properties (icing and bakery glazes, liquid and soft-texture food products, and edible films)	[23,24,26]
*Turbinaria* sp., *Ascophyllum* sp., *Durvillaea* sp., *Ecklonia* sp., *Laminaria* sp., *Lessonia* sp., *Mucrocystis* sp., *Sargassum* sp.	Alginate	Restructured meat and vegetable products, baked products, ice creams, frozen desserts	[23,24,26]
*C. crispus*	Carragenans	Gelling, emulsifying, thickening and stabilizing properties (ice cream, yogurt, cheese, milk-based products, bread, coating films)	[26]
*L. digitata*	Laminarin and Fucoidan	Increase lipid stability in pork meat products	[129]
*U. pinnatifida*	Fucoxanthin	Increase lipid stability in chicken products and enhance redness and yellowness in ground chicken breast meat	[130]
*P. palmata*	Protein hydrolisate	Functional (renin inhibition) bread with potential positive heart effects	[131]
*H. elongata*, *U. pinnatifida*, *P. umbilicalis*	PUFA	Seaweed addition increased n-3 PUFA but decreased n-6/n-3 PUFA ratio in pork products. Moreover, seaweeds conferred antioxidant activity and increased amino acid levels.	[132]
*F. vesiculosus*	Phlorotannins	Increased antioxidant activity in fish products	[133]

PUFA—polyunsaturated fatty acid.

**Table 3:** Feed applications of seaweeds.

Seaweed Species	Compounds	Applications	References
*Sargassum* sp., *Gracilaria* sp., *Gelidelia* sp., *Hypnea* sp.	Minerals, amino acids, carbohydrates	Feed for fish and prawn culture	[127]
*A. nodosum* (Tasco^®^)	Low protein content, high mineral content and large concentration of phlorotannins	Feed for ruminants. Increase resistance to stressors like mixing, livestock transportation, exposure to food-borne toxins, excessive heat or temperature; improvement of the immune system; increased productivity and/or quality of the animal (milk, meat, carcass grade, etc.); and reduction of risk of pathogen infection	[135,136,137,138]
*E. prolifera*	Mineral, polysaccharide and carotenoid contents	Improved egg production and quality; improved immune functions and intestinal microflora	[139]
*S. dentifolium*	UFA, PUFA, carotenoid contents	Decreased yolk cholesterol, triglycerides and n-6 fatty acids and increased carotenoid content	[140]

**Table 4:** Bioactivies of seaweeds with pharmaceutical and cosmetic applications.

Seaweed Species	Biological Activities	References
*A. tenera* and *N. fastigiata*	Antiviral activity	[147,148,149]
*C. socialis*, *S. latifolium*, *U. flexuosa*, *P. antillarum*, *P. boergeseni*, *U. reticulata*, *D. membranacea*, *U. flexuosa*, *C. vagabunda*, *U. lactuca*, *G. multipartita*, *C. glomerata*, *H. valentiae*, *Stigeoclonium* sp., *Ulothrix* sp., *Nitzschia* sp., *E. prolifera*, *U. rigida*	Antibacterial effects	[150,151,152,153,154,155,156,157,158,159,160,161,162]
*Fucus* sp., *Stypopodium* sp., *S. muticum*, *U. fasciata*, *Laminaria* sp., *Laurencia* sp., *I. okamurae*, *Lithothamnion* sp., *P. dentata*, *C. barbata*, *Lophocladia* sp., *A. nodosum*, *G. termistipitata*, *U. intestinalis*, *U. pinnatifida*	Anticancer activity	[163]
*U. fasciata, E. stolonifera, E. cava, E. maxima, E. bicyclis, I. okamurae, A. nodosum*, *S. hystrix, S. polycystum, P. boergesenii, P. tetrastromatica*, *F. vesiculosus, T. conoides*, *S. japonica, U. pinnatifida*, *P. pavonica,*	Antidiabetic effect	[164,165]
*H. valentiae*, *U. reticulata*, *E. stolonifera*, *I. okamurae*, *B. bifurcata*, *E. cava*, *E. bicyclis*	Neuroprotective effects	[9,166,167,168,169]
*U. lactuca*, *D. salina*, *C. tomentosum*, *U. rigida*, *A. nodosum*, *B. bifurcata*, *E. bicyclis*, *E. cava*, *E. stolonifera*, *F. vesiculosus*, *C. crispus*, *Gelidium* sp., *Gracilaria* sp.	Cosmetic applications (including anti-moisture, anti-ageing, anti-inflammatory, whitening effect, hair growth)	[170]

**Table 5:** Other applications of seaweeds.

Seaweed Species	Application	References
*Laminaria* sp., *Ascophyllum* sp., *Sargassum* sp.	Fertilizer	[127]
*M. pyrifera*, *Laminaria* sp., *Gracilaria* sp. and *Sargassums* sp.	Biomass for fuel (methane production)	[5,127]
*Sargassum* sp., *Laminaria* sp. and *Ecklonia* sp., *Ulva* sp. and *Enteromorpha* sp.	Wastewater treatment (accumulation of toxic metals)	[127]

## Data Availability

Not applicable.
